# ABC Assay: Method Development and Application to Quantify the Role of Three DWV Master Variants in Overwinter Colony Losses of European Honey Bees

**DOI:** 10.3390/v9110314

**Published:** 2017-10-27

**Authors:** Jessica L. Kevill, Andrea Highfield, Gideon J. Mordecai, Stephen J. Martin, Declan C. Schroeder

**Affiliations:** 1 School of Environment and Life Sciences, The University of Salford, Manchester M5 4WT, UK; J.kevill@edu.salford.ac.uk (J.L.K.); S.J.Martin@salford.ac.uk (S.J.M.); 2 Viral Ecology, Marine Biological Association, Plymouth PL1 2PB, UK; ancba@mba.ac.uk (A.H.); gmordecai@eoas.ubc.ca (G.J.M.); 3 Department of Earth, Ocean and Atmospheric Sciences and Biodiversity Research Centre, The University of British Columbia, Vancouver, BC V6T 1Z4, Canada; 4 School of Biological Sciences, University of Reading, Reading RG6 6LA, UK; 5 Veterinary Population Medicine, College of Veterinary Medicine, University of Minnesota, St Paul, MN 55108, USA

**Keywords:** deformed wing virus, quasi-species, honey bees, RT-qPCR, overwintering colony loss, superinfection exclusion

## Abstract

Deformed wing virus (DWV) is one of the most prevalent honey bee viral pathogens in the world. Typical of many RNA viruses, DWV is a quasi-species, which is comprised of a large number of different variants, currently consisting of three master variants: Type A, B, and C. Little is known about the impact of each variant or combinations of variants upon the biology of individual hosts. Therefore, we have developed a new set of master variant-specific DWV primers and a set of standards that allow for the quantification of each of the master variants. Competitive reverse transcriptase polymerase chain reaction (RT-PCR) experimental design confirms that each new DWV primer set is specific to the retrospective master variant. The sensitivity of the ABC assay is dependent on whether DNA or RNA is used as the template and whether other master variants are present in the sample. Comparison of the overall proportions of each master variant within a sample of known diversity, as confirmed by next-generation sequence (NGS) data, validates the efficiency of the ABC assay. The ABC assay was used on archived material from a Devon overwintering colony loss (OCL) 2006–2007 study; further implicating DWV type A and, for the first time, possibly C in the untimely collapse of honey bee colonies. Moreover, in this study DWV type B was not associated with OCL. The use of the ABC assay will allow researchers to quickly and cost effectively pre-screen for the presence of DWV master variants in honey bees.

## 1. Introduction

RNA viruses have high mutation rates and exist as a diverse population of variants or quasi-species [1]. Quasi-species are a range of variants, genetically linked through mutation and organised around a master sequence or variant [2,3]; they undergo a constant process of mutation, competition, and selection [4]. This provides a cloud of variants with an evolutionary advantage, allowing them to occupy several biological niches; however, even a single amino acid change can completely alter the pathogenicity of a virus [4].

Of the many RNA viruses that infect the European honey bee, the acute bee paralysis virus (ABPV) and deformed wing virus (DWV) quasi-species complexes are readily detected in asymptomatic bees and often associated with colony losses [5,6]. It is proposed that ABPV, Israeli acute paralysis virus (IAPV), and Kashmir bee virus (KBV) all belong to the same cloud of ABPV variants [5,6]. ABPV follows a classic acute-type infection strategy that rapidly translates into overt symptoms of paralysis and ultimately death for the honey bee. Virulence is highly dependent on the mode of transmission and type of the genetic variant; for example, the differences in pathology among different strains of IAPV found globally is likely due to high levels of standing genetic variation [7].

Deformed wing virus (DWV), on the other hand, is a one of an increasing number of emerging RNA viral pathogens that are capable of replicating in a wide range of invertebrate species, such as; bumblebees, wasps, hornets, ants, hoverflies, and solitary bees [8,9]. More importantly, DWV is also vectored by ectoparasitic mites of honey bees [10,11,12]. Previous analysis indicates that the DWV population consist of many different variants [5,13,14]. When DWV infection is associated with the mite *Varroa destructor* there is a dramatic increase in viral load [14,15] and a loss in DWV diversity [13,16], which is associated with the death of the honey bee colony. Therefore, DWV exists as a quasi-species and is now the most regularly detected virus in honey bees.

Considering DWV’s host range and varying impact on different honey bee populations and hosts, it is important to establish the role that each DWV master variant within the various insect communities play. Currently, three master variants of DWV have been identified: Type A [15], B [17], and C [14]. Type A is the most prevalent and has been linked to colony declines [13,18,19]. DWV type B (previously designated as Varroa destructor virus-1 (VDV-1) has been implicated in the protection of the colony against DWV type A in a mite infested honey bee population in the UK [20], although type B can be pathogenic at the level of the individual honey bee [21]. The effects of DWV type C are still unknown, as it is the most recently-described variant [14]. Furthermore, the ability of the three master variants to infect the same host provides the opportunity for viral recombinants of DWV to form and a range of recombinants have been detected for DWV master variants [14,22,23].

The most common detection method for DWV is reverse transcriptase polymerase chain reaction (RT-PCR), the majority of primers used are targeting the *RNA dependent RNA polymerase gene* (*RdRp*) [19,21,24,25,26,27]. Primers which target other regions in the DWV genome such as the *Lp*, *Helicase*, *VP1*, and *VP2*, have also been described [22,23,28]. Using several regions allows for the identification of DWV recombinants. With the exception of McMahon et al. and Moore et al., current studies do not discriminate between Type A or B [13,18,24,25,26,27,28,29,30] and have concentrated upon its prevalence and load, rather than the variants present or only report on one master variant. High resolution melt analysis (HRM) was previously used post RT-PCR to identify variations in nucleic acid sequences by detecting differences in amplicon dissociation curves [14,22]. Martin et al. reported the separation of DWV type A and B via HRM. Unfortunately, DWV type C fell within the same dissociation curves as DWV type B [14]. To our knowledge, no assay has been designed to report on all three known master variants of DWV.

More recent quantitative-PCR (qPCR) methods have been developed, which allows for DWV viral loads to be calculated by either absolute quantification using a DNA [21] or RNA [25] standards or via relative quantification using a ∆*C*_t_ (delta critical threshold) value determined relative to a known housekeeping gene [11,19,26,27,30]. The absolute quantification method is considered the “gold standard” as it provides virus genome equivalent data which, in turn, can also be normalised to housekeeping genes for between-sample comparisons (e.g., [25]); and it can, in the long run, save both money and time [31]. Research efforts are now moving towards incorporating next generation sequencing (NGS), which produces a vast amount of data pertaining to viral variants, recombinants, and load [14,22,23,32,33]. However, this technique is both costly and time-consuming. 

As evidenced, a simple DWV presence/absence assay using RT-PCR has limited value as the realisation that the viral load and variant diversity is vital in studying the evolution and impact of DWV master variants upon pollinator health. Therefore, primers which can detect all of the major DWV variants (A, B, and C) are required as a first step to investigate the impact that each DWV master-variant has upon its hosts and how they are evolving or co-evolving. Here we report on a RT-qPCR or ABC assay, based on new set of primers, to detect all the known DWV master variants (A, B, and C). This will hopefully allow researchers around the globe to investigate the role of the various variants via a common method, thus allowing the results generated across the various studies to be comparable. We applied the assay to archived material collected during the Highfield et al. overwintering colony loss (OCL) study, which reported that the relative increase in DWV load during the overwintering period was implicated in colony losses. Here we provide evidence that multi-DWV master variant type A and C are responsible for the decline in colony health. This is the first time in which type C has been recorded in dying colonies, with the implication that the low levels of DWV type B were unable to protect the colony [20].

## 2. Materials and Methods

### 2.1. Primer Design

Primers were designed using the DWV reference sequences for DWV type A (NC_004830.2), type B (AY_251269.2) and type C (CEND01000001.1). The primers were designed in a similar *RdRp* region used by Highfield et al., as this is a highly-conserved region of the genome. All three primers use the same forward primer binding site since it has a high identity within, and between, master variants, and three different reverse primers (Table 1). Primers were designed with similar thermodynamic properties to allow each to be used in the same PCR cycling conditions.

### 2.2. Viral Master Variant Plasmid Standards

To create standards for each of the three primers, plasmid vectors containing the target genes (*RdRp* region) of DWV master variants type A, B, and C were created (Biomatik, Cambridge, ON, Canada, Table 2) and reconstituted as per the manufacturer’s guidelines. Heat shock transformation was performed using One Shot^®^ TOP10 chemically-competent *Escherichia coli* following the manufacturer’s instructions (Life technologies, Carlsbad, CA, USA). Using an aseptic technique, the transformants were selected on LB agar plates, containing kanamycin and left to incubate at 37 °C overnight. A single colony for each master variant was propagated in LB medium containing kanamycin and a Qiaprep^®^ spin mini prep kit (Qiagen, Manchester, UK) was used per the manufacturer’s instructions to extract plasmid DNA. The resultant plasmids were diluted to 56 ng/μL and used in PCRs targeting the M13 regions flanking the multiple cloning sites (Table 1); sequences were confirmed and used to produce linear strands of DNA which served as templates in PCR assays. M13 PCR products were visualised on 1% agarose gel.

### 2.3. Primer Optimisation

Temperature step gradient PCR (48, 50.5, 52.9, 56.1, 58.5, and 61 °C) was performed to assess which annealing temperature provided the most specific result, using the newly-designed primers listed in Table 1. Here, each PCR reaction contained 2 μL plasmid DNA, 10 μL 5× buffer, 5 μL MgCI_2_, 5 μL dNTPs 2.5 mM, 2 μL DWV-forward primer (10 pmol), 2 μL DWV reverse primer (10 pmol) (A, B, or C, Table 1), 0.2 μL GoTaq^®^ G2 Flexi DNA polymerase (Promega, Madison, WI, USA), and 23.8 μL RNAse free H_2_O. The results were visualised on 2% agarose gel.

### 2.4. Viral Master Variant cRNA Standards

To synthesise cRNA, 100 ng of gel purified M13 linear PCR product was recovered using a Zymoclean™ gel DNA recovery kit (Zymo research, Irvine, CA, USA) and quantified using an Agilent 2200 TapeStation (Agilent, Santa Clara, CA, USA). RNA transcription was conducted in vitro using a mMessage mMachine^®^ T7 Kit (Life technologies, Carlsbad, CA, USA), following the manufacturer’s instructions. Samples were treated with Turbo™ DNase (Life technologies, Carlsbad, CA, USA) to remove DNA. cRNA recovery was conducted using an Ambion MEGAClear™ Kit (Life technologies, Carlsbad, CA, USA).

### 2.5. Method Validation

Quantification of nucleic acids (plasmid DNA and cRNA) for DWV variants A, B, and C was conducted using an Agilent 2200 TapeStation prior to analysis. To test reaction efficiency and create true competition experiments between primer sets for each of the DWV variant targets, a 10-fold dilution series and sample mixes containing each DWV variant in different concentrations were made from the quantified plasmid DNA and cRNA. Analyses were conducted via real-time PCR and real-time RT-PCR, respectively, on a Rotor-Gene 6000 (Qiagen, Manchester, UK). In addition, competitive real-time qPCR and real-time RT-qPCR was also conducted on each DWV variant for both plasmid DNA and cRNA, respectively, i.e., the amplified *C*_t_ value for each variant were compared against a DNA or RNA standard curve. The mixed samples were analysed against a standard curve. Each primer pair was also tested sequentially against all three DWV variants, to ascertain if non-specific amplification occurred. Three master mixes were prepared for each DWV reverse primer (Table 1). The reactions for DNA contained 1 μL DNA, 10 μL SensiFAST™ SYBR^®^ No-Rox (Bioline, London, UK), 0.75 μL DWV forward primer, 0.75 μL reverse primer (Table 1) and 7.5 μL RNAse free H_2_O. Initial activation occurred at 95 °C for 3 min, followed by 35 cycles of denaturing at 95 °C for 15 s, annealing at 58.5 °C for primers A and B, and 61.5 °C for primer C for 15 s, and extension at 72 °C for 15 s.

RT-PCR was performed on the RNA using a SensiFAST™ SYBR^®^ No-Rox One Step kit (Bioline, London, UK). Each reaction contained 10 μL SensiFAST™ SYBR^®^ No-ROX One-Step mix (2×), 0.75 μL DWV forward primer, 0.75 μL reverse primer (A, B or C; Table 1), 0.2 μL reverse transcriptase, 0.4 μL RiboSafe RNase Inhibitor, 7.5 μL RNAse free H_2_O. The RT step occurred at 45 °C for 10 min and denaturation at 95 °C for 10 min, followed by 35 cycles of denature 95 °C 15 s, annealing at 58.5 °C for 15 s and extension at 72 °C for 15 s. RT negative PCR was also performed on the cRNA to confirm the absence of DNA in the cRNA.

Robustness of the RT-PCR assay was also determined by spiking each target variant with equimolar concentrations at 9.94 ng/μL or 10-fold lower concentrations of competing master variant cRNAs. This was done to determine whether competing RNA could affect the linear range of the assay. The mean *C*_t_ values (carried out in triplicate) plus standard deviation was calculated.

For both cRNA and DNA experiments, a melt curve analysis was performed between 72 °C and 90 °C, at 0.1 °C increments, each with a 5 s hold period. Purity and amplicon size was confirmed on 2% agarose gel. Both methods ensured that no contamination was present in the negative template controls and that one product was amplified per primer set. Each sample (plasmid DNA and cRNA) was analysed in duplicate. Copy numbers were determined using the following equations:

Copy number plasmid DNA = (Concentration of DNA (ng/µL) × 6.022 × 10^23^) (1)

(Fragment length base pairs × 109 × 650)(2)

Copy number RNA = (Concentration RNA (ng/µL) × 6.022 × 10^23^) (3)

(Fragment length base pairs × 109 × 325)(4)

### 2.6. Application of the RT-qPCR (ABC Assay)

Honey bee samples, which had previously tested positive for DWV [25,34] and had NGS data available [14,34], were selected for analysis. The 11.6, 6.7, and 21 million reads retained after BLASTn (Basic Local Alignment Search Tool) hits to a DWV reference database for samples GD1 June, GD1 late Oct and OW1a, respectively, were further partitioned by mapping the reads to the three DWV master variant genome [14,33]. The honey bee samples (GD) were collected throughout 2006–2007 from Devonshire locations, and in 2015 from Hawaii (OW), these samples were archived at −80 °C. A 30 mg sub-sample (representing ~2% of the total biomass) of previously-pooled and ground honey bees were weighed before total RNA was extracted using an RNeasy^®^ mini kit (Qiagen, Manchester, UK) following the manufacturer’s instructions. Quantification of the total RNA was established using a spectrophotometer (Nanodrop 2000, Thermo Fisher Scientific, Wilmington, DE, USA). RNA was diluted to 50 ng/μL to avoid concentration-dependent effects on RT-qPCR efficiency [35]. The ABC assay was performed in triplicate for each of the DWV variants and actin. An actin control was deemed necessary to assess levels of degradation due to the long-term storage of samples. Master mixes were made, for each of the DWV primer sets, using a Sensifast™ SYBR^®^ No-Rox One Step Kit (Bioline, London, UK), and the temperature profile previously detailed. Genome equivalents were calculated per bee using the following equation:

Genome equivalents = (average copy number) × (RNA dilution factor) × (elution volume of RNA) × (proportion of bee material)(5)

### 2.7. HRM Assay

High-resolution melt (HRM) analysis was conducted upon the archived Devonshire bee samples as per the protocol described in Martin et al. [10].

### 2.8. Sequencing

A selection of the PCR or qPCR products generated in method validation (Section 2.2, Section 2.3, Section 2.4 and Section 2.5) and application of the ABC assay (Section 2.6) were Sanger sequenced [10] to confirm the specificity of the assay.

## 3. Results

### 3.1. PCR and RT-PCR Sensitivities 

The sequencing results of the three DWV variant plasmids confirmed that they could be used in the PCR assays and for cRNA synthesis for the eventual use in the RT-PCR assays (Table 2). The optimum annealing temperature (as defined by the tightest PCR band of the expected size produced on an agarose gel without the presence of other fainter non-specific multiple sized bands) for the three primer sets was determined to be 58.5 °C when using plasmid DNA and cRNA as a template. Interestingly, when honey bee total RNA was used as a template the annealing temperature needed to be increased to 61.5 °C, especially when amplifying type C.

### 3.2. Competitive PCR

**Figure 1:**
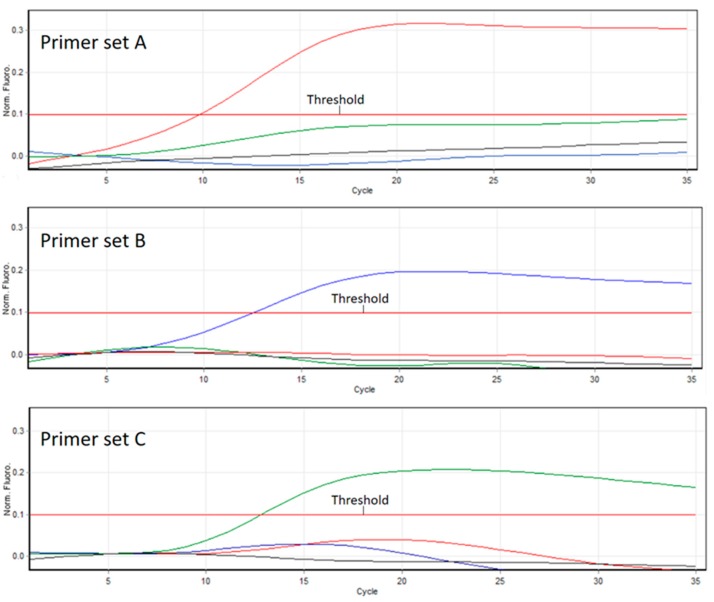
Amplification plot of competitive PCR. Red line = Type A, blue line = Type B, green line = Type C, and black line = no template control. All primer sets amplified the target DWV variant, and non-targets are represented by coloured lines under 0.1 fluorescence.

Competitive PCR confirmed primer sets were specific to each DWV variant and amplification of non-targets did not occur (Figure 1). This was further confirmed through RT-PCR melt curve analysis (Figure S1A) and sequencing (Figure S1B). 

### 3.3. PCR and RT-PCR Efficiencies

**Figure 2:**
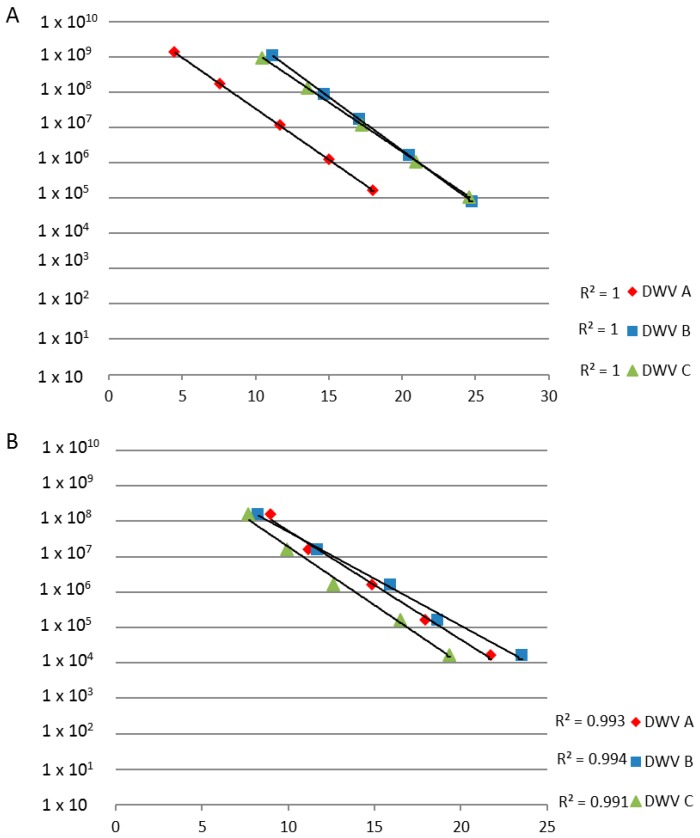
Ten-fold dilution series for cRNA (**A**) and plasmid DNA (**B**) using the RT-PCR and PCR assays; the standard deviation bars are not shown for clarity.

The performance of each primer set targeting the DWV *RdRp* region in a dilution series reveals that all the reactions were between 99% and 100% efficient (Figure 2). Interestingly, the RT-PCR (R^2^ = 1, −3.3 slope) was marginally more efficient than the PCR (R^2^ = 0.99, −3.5 slope), however, the sensitivity of the reactions differ greatly within and between the RNA and DNA based assays. The cRNA standards (10^9^ to 10^3^ copies) detected in the *C*_t_ range spanning five to 25 cycles, while the PCRs starting with plasmid DNA as the template (10^8^ to 10^4^ copies) fell within the *C*_t_ range of between 8 and 25 *C*_t_ values (Figure 2). Primer set A was more sensitive and quantification can be carried between 4.5 and 25 *C*_t_ cycles whereas, for primer sets B and C, the *C*_t_ range is between 0 and 25 *C*_t_ cycles. The maximum number of cycles achievable for both RT-PCR and PCR were 35 cycles (=*C*_t_ 30) before non-specific and background cross-contamination could be detected. PCRs containing less than 10^3^ and 10^4^ copies of cRNA and plasmid DNA, respectively, fell outside of the range of quantification.

**Figure 3:**
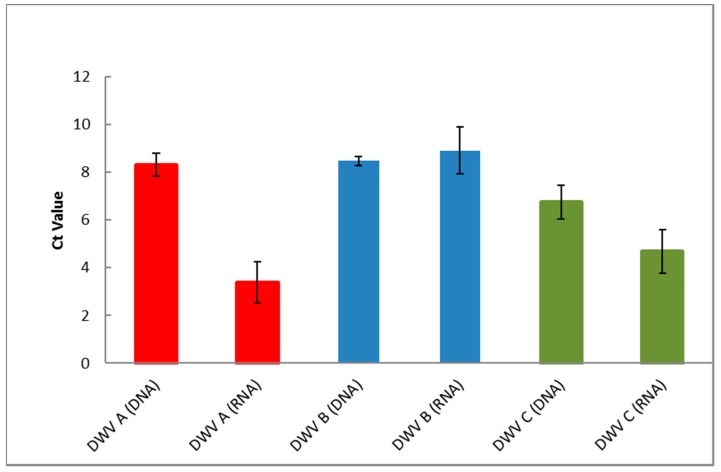
Reproducibility of RT-PCR and PCR assays for each DWV primer set. Bars display the mean *C*_t_ of the average data calculated from the dilution series. Error bars show the deviation from the mean *C*_t_ between serial dilutions. DNA from plasmid, RNA refers to cRNA, DNA copy number = 10^9^, RNA copy number = 10^10^.

Both RT-PCR and PCR assays within and across different dilutions were highly reproducibility (Figure 3). The mean Ct value for primer set A, B, and C detecting 10^9^ copies of plasmid DNA being 8 (± 0.49), 8 (± 0.20) and 7 (± 0.70), respectively. This was different for the cRNA which had a mean *C*_t_ for primer sets A, B, and C of 3 (± 0.85), 9 (± 0.97), and 5 (± 0.91), respectively. Variation of <1 *C*_t_ from the average *C*_t_ value was witnessed for both RNA and DNA, therefore, reactions containing both RNA and DNA are highly reproducible (Figure 3).

### 3.4. Robustness of the RT-qPCR (ABC Assay)

**Figure 4:**
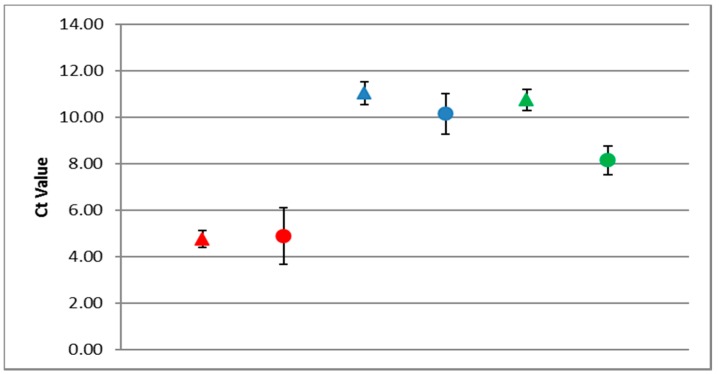
ABC assay. Red = DWV type A, blue = DWV type B, and green = DWV type C. Triangles represent DWV target only, and circles represent DWV samples containing multiple variants. Error bars represent the standard deviation from the mean *C*_t_.

*C*_t_ values varied from the standard curve (triangles, Figure 4) when samples contained multiple master variants (circles, Figure 4). A 3.3 *C*_t_ value deviation is the equivalent of a 10-fold increase/decrease in copy number, the deviation in this assay for primer set A is equal to 1.2 *C*_t_’s, B = 0.87, and C = 0.61. Therefore, multiple variants present in the sample can affect the reaction stability but by no more than a three-fold increase/decrease in copy number.

### 3.5. Detection of DWV in Honey Bees Using the ABC Assay

**Figure 5:**
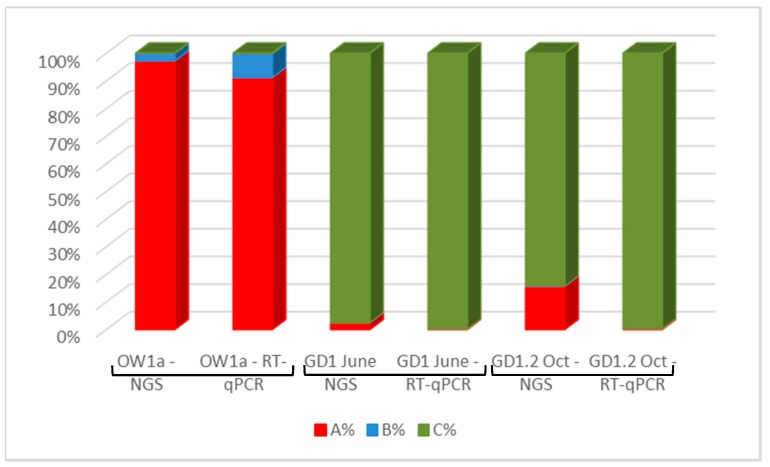
Comparison of ABC assay and NGS data for honey bees. Red = DWV variant A, blue = DWV variant B, and green = DVW variant C.

The results of RT-qPCR (ABC assay) revealed that all three master DWV variants were detected in honey bee samples (Figure 5). To further confirm the specificity of the new primers, the ABC assay data was compared to NGS data obtained for the same samples (Figure 5). Both assays detected the same DWV variants; a further confirmation of the specificity of ABC assay. The number of reads and viral loads differ between the results of the NGS and ABC assay, respectively, however, the presence of similar variants in each sample, and also that the sample was dominated by the same master variant, was consistent across all samples.

### 3.6. DWV Master Variants Implicated in OCL

**Figure 6:**
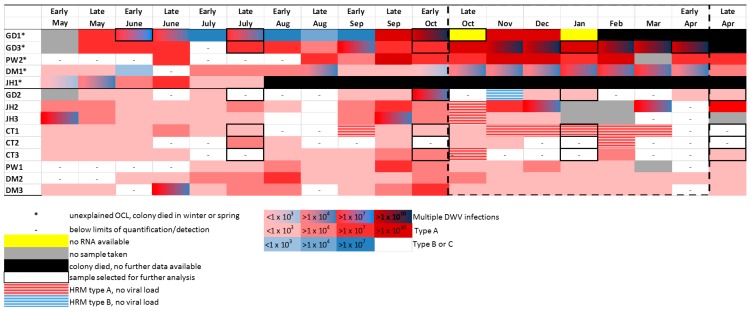
DWV load plus variant data as revealed by HRM for bee colonies sampled in Devon, in the southwest of England (Shute, Honiton, Plymouth, Ashburton, and Newton Abbot), over a year (bimonthly between May and October 2006, monthly between November 2006 and March 2007, and bimonthly in April 2007) as described in the Highfield et al. study [21]. The DWV variants are displayed by colours: red = DWV type A, blue = DWV type B or C, and the shade of the colour relates to the viral load; light colours represent low viral loads, dark colours represent high viral load.

A study carried by Highfield et al. resulted in the collection of asymptomatic worker honey bees from three separate hives per apiary from a total five apiaries (*n* = 15 colonies), all known to have a history of Varroa mite infestation [25]. They were sampled over a year (bimonthly between May and October 2006, monthly between November 2006 and March 2007, and bimonthly in April 2007). All colonies with Varroa mites underwent control treatments to ensure that mite populations remained low throughout the study. Despite this, multiple virus infections were detected, yet a significant correlation was observed only between DWV viral load and overwintering colony losses (OCL). Our current dataset based on the HRM analysis (Figure 6, Figure S2) reveal that multiple infections with DWV master variants coupled with consistently high viral loads (>10^7^ DWV genome equivalents per honey bee) for several consecutive winter months played a causative role in OCL [25] observed in colonies GD1, GD3, PW2, and DM1 (Figure 6). Conversely, multiple DWV master variant infections were less frequent and at relatively lower levels (<10^7^ DWV genome equivalents per honey bee) during the winter months in the colonies that survived [25] the winter (Figure 6).

The ABC assay was applied to select samples, focusing on months which represent the summer (July), autumn to winter transition (October) and winter months (January). The first spring bees (April) were also screen for the colonies that survived the overwintering period (Figure 6 and Table 3). DWV master variants type A and C were detected in the majority of samples screened (Table 3), type B was infrequent and up to three orders of magnitude fewer in viral load when compared to the other two master variants, especially in the colonies that suffered OCL. In addition, of the colonies which collapsed, type C was the dominant variant in the final month before collapsing, reaching 10- to 100-fold more that the next highest variant. The ABC assay was only two orders of magnitude more sensitive that the HRM assay.

DWV type A was consistently present in all the samples tested. Interestingly, of the colonies that collapsed, GD1 and GD3 reported mid-winter highs (January) of >10^10^ DWV genome equivalents per honey bee (Table 3). This is in contrast to summer (6 July) samples which revealed through NGS (Figure 5) and the ABC assay (Table 3), respectively, to have been present in only a tiny proportion of type A (0.002087% of the total 10^7^ DWV infection in GD1, 6 July) (Table 3). We note that it is at this time that DWV type C starts to dominate the DWV variant landscape (Table 3). The role of DWV type C is less clear in GD3. Nonetheless, both DWV variants type A and C dominated over type B in the colonies which experienced OCL.

## 4. Discussion

The PCR assay that forms the basis of the ABC assay was shown to be specific for the detection of each DWV variant with no observed cross-reactivity, as evidenced via competitive PCR (Figure 1). We also go on to show the importance of using RNA, as opposed to DNA, in standards in RT-PCR-based assays when quantifying RNA viruses (Figure 2 and Figure 3).

As expected, the PCR efficiencies were the same for both PCR and RT-PCR assays, confirming the doubling effect of the product during each PCR cycle. Deviations were nonetheless observed in *C*_t_ values (Figure 4) when multiple DWV master variants were present in a sample. These deviations are considered to be caused by the forward primers being targeted by all the master variants in the reaction. In addition, as RNA viruses exist as a constantly mutating cloud of similar variants it is impossible to predict the role of unknown variants upon the RT-PCR assay. Nonetheless, the accuracy and robustness of the RT-PCR assay appears to be within a 0.5- to two-fold level (Figure 4).

A comparison of the ABC assay with the NGS data confirms the efficacy of the assay. Discrepancies were observed between the precise amounts of DWV variant genome equivalents present; however, both methods yielded similar results, i.e., showing one dominant master variant over the less dominant master variant. These discrepancies are considered to be caused, firstly, by the NGS data obtained from oligo dT priming with a 3′ amplification bias. Given that the *RdRp* target is in this 3′ region [23], the inefficiency of the RT step could account for minor differences. Secondly, as we compared the output of the top hit BLAST (Basic Local Alignment Search Tool) analysis from all the reads across the whole genome, samples confirmed to be free or with low-level recombinants, whereas our ABC assay is reporting only on the *RdRp* region. Thirdly, different methodologies will have different sensitivities. Nassirpour et al. [36] conducted a comparative study between NGS and RT-qPCR and found minimum agreement between NGS and RT-qPCR platforms. They conclude that RT-PCR is more sensitive due to the use of targeted specific primer pairs.

The HRM analysis revealed multiple DWV variants present in the Devonshire bees. The HRM technique can only separate DWV type A from type B-C master variant mix, with type B and C sharing similar melting temperatures [13]. However, analysis using the ABC assay revealed that type C was the most prevalent variant for DWV melt curves falling within the type B or C melt temperature range (<77.5 °C) [13]. Type B was infrequent and was present at lower levels independent on whether the colony collapsed or not. This result supports the superinfection exclusion hypothesis proposed by Mordecai et al. (2016), who suggest that type B is less virulent than other master variants of the virus [20]. Here we hypothesize that it was not in sufficiently high enough numbers to protect colonies from the more virulent type A, and possibly C, variants. This is the first time that type C has been shown to be present, although not exclusively so, in colonies which collapsed during the winter period, and highlights the importance of variant specific assays for DWV detection. Given the sample size of both the Swindon [20] and Devon [25] studies, further validation of the virulence of type C and putative protective nature of type B (through superinfection exclusion) is still required. Nonetheless, this study clearly shows how the ability to track variants throughout the season, at various sampling points, provides the opportunity for researchers to assess the putative role of DWV viral competition and evolution in honey bees. The HRM data revealed that DWV infection is variable between sampling points, as expected with RNA viruses which are under a constant state of competition [1].

The development of the DWV ABC assay provides an opportunity for a consensus approach to DWV research efforts. Standard curves are considered laborious to develop [37] and primer design can be troublesome, as efficiency and specificity must be equal to avoid poor reactions [38]. Past studies focused on the effects of multiple viruses upon colony health [25,30,31,32,33,34,35,36,37,38,39,40,41,42,43] that have revealed the role of DWV as a major factor in colony losses. Now, research efforts must focus on the effects of DWV variants. Currently, the knowledge about variant diversity upon honey bee health is limited, in particular for the type C variant. Using three distinct primer sets provides an opportunity to study the effects of DWV variant diversity upon honey bee health for each of the known master variants of DWV. The ABC assay allows for a quick, reliable, robust, and quantitative method of three known master variants of DWV, providing an insight into total DWV infection in honey bees.

## Figures and Tables

**Table 1:** Primers used in this study.

Target	Primer Name	Sequence (5′–3′)	Genome Target (NC004830.2)	Size of Product (bp)	Reference
DWV	DWVnew-F1	TACTAGTGCTGGTTTTCCTTT	8653–8673		This study
DWV Type A	DWVA-R1	CTCATTAACTGTGTCGTTGAT	8808–8788	155	This study
DWV Type B	DWVB-R1	CTCATTAACTGAGTTGTTGTC	8808–8788	155	This study
DWV Type C	DWVC-R1	ATAAGTTGCGTGGTTGAC	8805–8788	152	This study
DWV q	DWVq-R1	CTGTGTCGTTGATAATTGAATCTC	8656–8676	145	[25]
	DWVq-F1	TAGTGCTGGTTTTCCTTTGTC	8800–8777		
M13	M13F	GTAAAACGACGGCCA	Na	361	[25]
	M13R	CAGGAAACAGCTATG			
Actin	ActinR1	AAGAATTGACCCACCAATCCATAC	Na	120	[25]
	ActinF1	CCTGGAATCGCAGATAGAATGC			

**Table 2:** DWV master variant (A, B, C) insert used in the plasmids. Primer sequences are underlined and reverse primer sequences are highlighted in bold.

Target	Insert
DWV A	TATCTTGGAATACTAGTGCTGGTTTTCCTTTGTCTTCATTAAAGCCACCTGGAACATCAGGTAAGCGATGGTTGTTTGATATTGAGCTACAAGATTCGGGATGTTATCTCTTGCGTGGAATGCGTCCCGAACTTGAGATTCAATT**ATCAACGACACAGTTAATGAG**GAAAAAGGGA
DWV B	TATCCTGGAATACTAGTGCTGGTTTTCCTTTATCTTCATTAAAACCGCCAGGCTCTTCTGGTAAGCGATGGTTGTTTGATATTGAATTACAAGATTCAGGATGTTATCTTTTGAGAGGGATGAGACCTGAACTTGAGATACAGTT**GACAACAACTCAGTTAATGAG**GAAGAAGGGA
DWV C	TTTCGTGGAATACTAGTGCTGGTTTTCCTTTATCCTCACTGAAACCAGCTGGAACATCAGGAAAAAGGTGGTTATTTGATATTGAATTGCAAGATTCGGGATGTTATCTTTTACGAGGTATGCGTCCCGAATTAGAAATACAATT**GTCAACCACGCAACTTAT**GAGGAAAAAGGGA

**Table 3:**
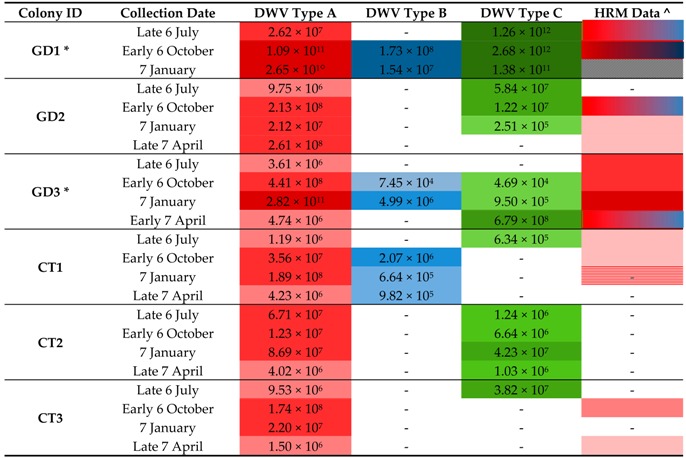
ABC assay data alongside its respective HRM analysis on a reduced dataset (Figure 6). DWV variant type and viral loads (darker shades indicated relative high viral loads) are displayed for each sample.

* Unexplained OCL, colony died in winter or spring; ^ taken from Figure 6; - below limits of quantification/detection; 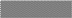: no RNA available; 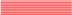: HRM type A, no viral load; 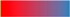: Multiple DWV variants; 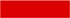: DWV type A; 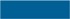: DWV type B (in ABC assay only); 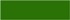: DWV type C.

## Supplementary Materials

The following are available online at www.mdpi.com/1999-4915/9/11/314/s1, Figure S1: validation of specificity of target amplification through (A) Melt curve analysis and (B) sequencing of resultant PCR product, Figure S2: HRM melt curves of monthly samples from colonies that (A) GD1 = collapsed during the over-wintering period, i.e., over-wintering colony loss (OCL) and (B) GD2 = survived the following year, i.e., healthy colony. Shaded area indicates the melt position of the type B or C variants in the months may to Sep in the OCL colony.
