# Supplementary material for: ABC Assay: Method Development and Application to Quantify the Role of Three DWV Master Variants in Overwinter Colony Losses of European Honey Bees
Source: Viruses. 2017 Oct 27;9(11):314. doi: 10.3390/v9110314 (PMC5707521; doi:10.3390/v9110314)
Supplement: Supplementary file 1 [file viruses-09-00314-s001.pdf]

A

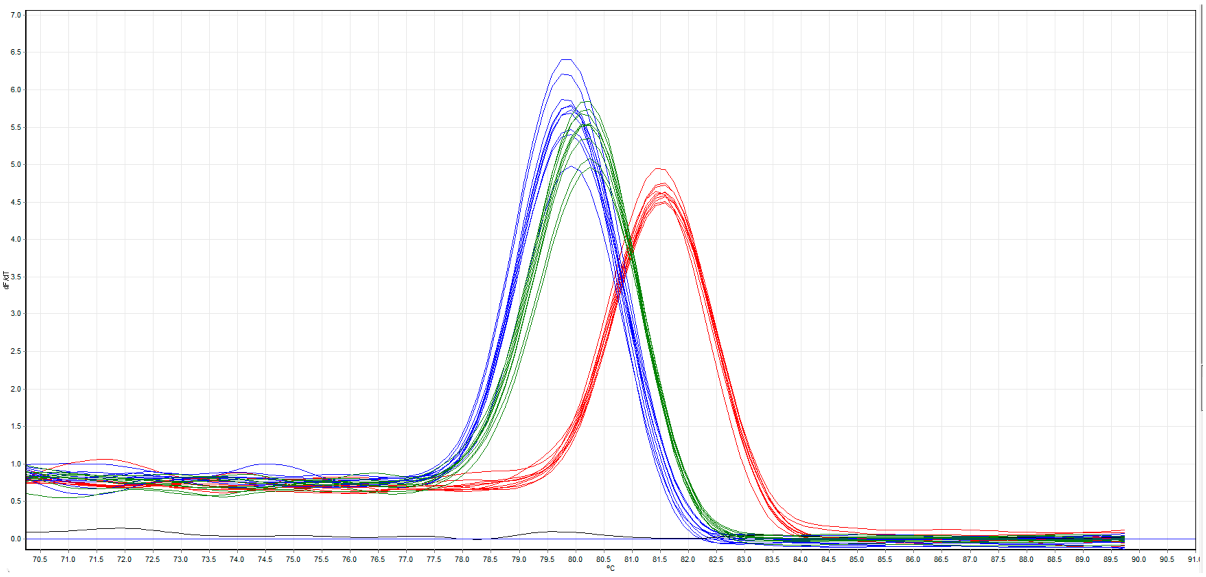

B

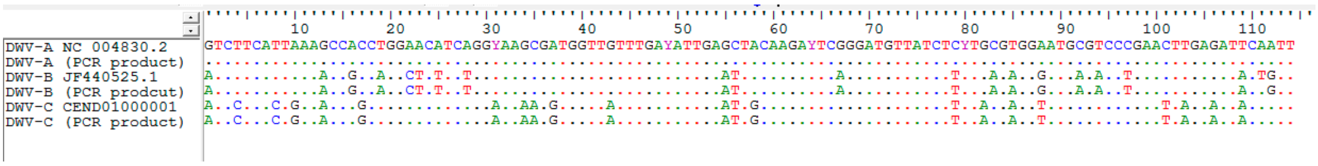

Figure S1: Validation of specificity of target amplification through A) Melt curve analysis and B) sequencing of resultant PCR product.

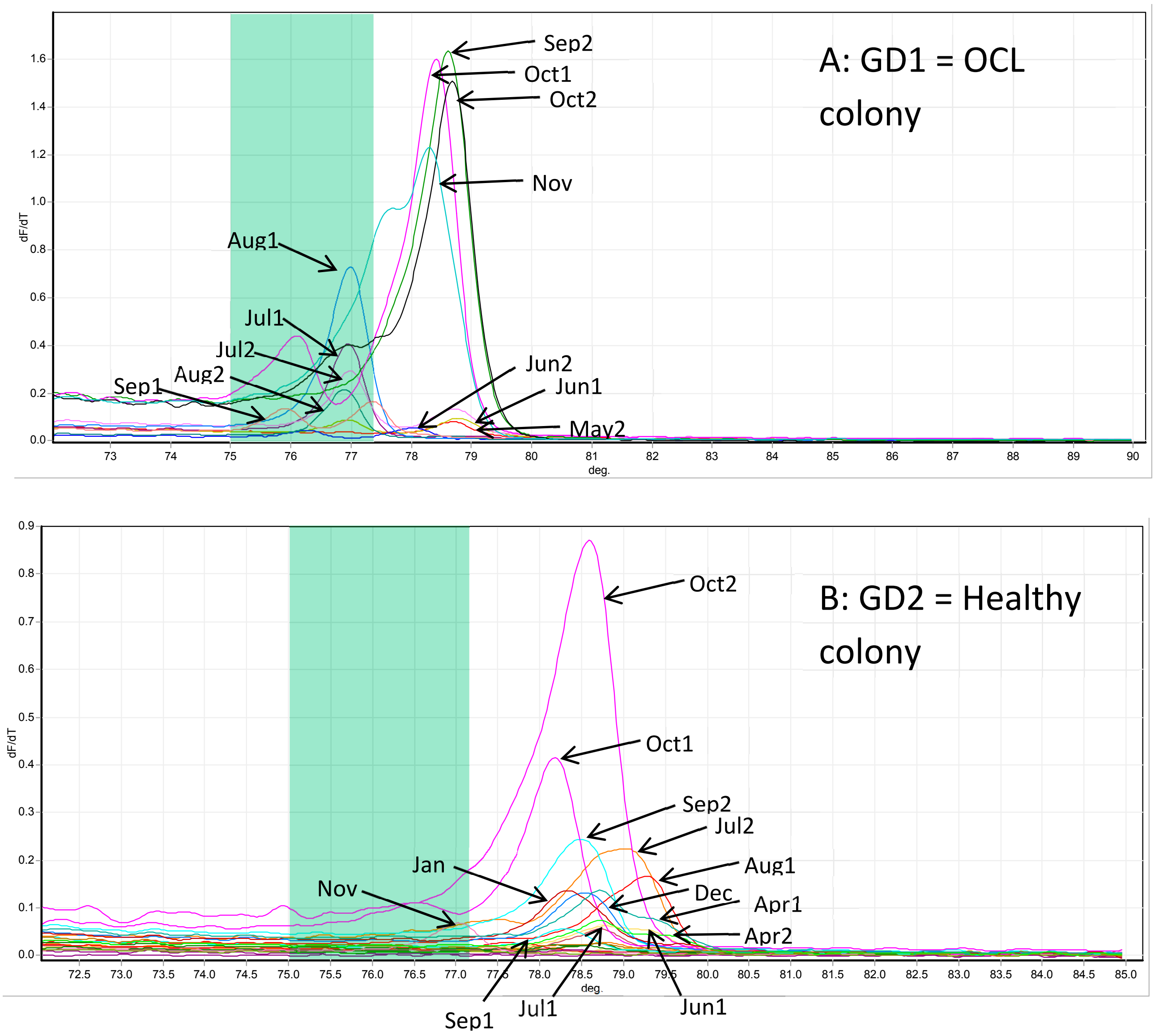

Figure S2 HRM melt curves of monthly samples from colonies that A) GD1 = collapsed during the over-wintering period, i.e. over-wintering colony loss (OCL) and B) GD2 = survived the following year, i.e. healthy colony. Shaded area indicates the melt position of the type B or C variants in the months may to Sep in the OCL colony.
